# Extended-Spectrum ß-Lactamase-Producing *Escherichia coli* Among Humans, Beef Cattle, and Abattoir Environments in Nigeria

**DOI:** 10.3389/fcimb.2022.869314

**Published:** 2022-04-07

**Authors:** Mabel Kamweli Aworh, Eme Ekeng, Pernille Nilsson, Beverly Egyir, Christian Owusu-Nyantakyi, Rene S. Hendriksen

**Affiliations:** ^1^ Department of Veterinary and Pest Control Services, Federal Ministry of Agriculture and Rural Development, Abuja, Nigeria; ^2^ Nigeria Field Epidemiology and Laboratory Training Program, Abuja, Nigeria; ^3^ National Reference Laboratory, Nigeria Center for Disease Control, Abuja, Nigeria; ^4^ Technical University of Denmark, National Food Institute, WHO Collaborating Centre (WHO CC) for Antimicrobial Resistance in Foodborne Pathogens and Genomics, FAO Reference Laboratory (FAO RL) for Antimicrobial Resistance, Europea Union Reference Laboratory for Antimicrobial Resistance (EURL-AMR), Kongens Lyngby, Denmark; ^5^ Department of Bacteriology, Noguchi Memorial Institute for Medical Research, College of Health Sciences, University of Ghana, Accra, Ghana

**Keywords:** antimicrobial, resistance, abattoir, cattle, environment, *Escherichia coli*, Nigeria

## Abstract

**Introduction:**

Beef cattle, one of the food-producing animals, are linked to humans through a shared environment and the food chain as a major source of animal protein. Antimicrobial drugs are readily accessible for use in food animal production in Nigeria. Beef cattle and abattoir environments harbor pathogenic bacteria such as *Escherichia coli* (*E. coli*) which have developed resistance to antimicrobial agents used for prophylaxis or treatment. This study investigated the zoonotic transmission of extended-spectrum beta-lactamase-producing *E. coli* (ESBL-EC) among humans, beef cattle, and abattoir environments in Abuja and Lagos, Nigeria.

**Materials and Methods:**

We conducted a cross-sectional study among abattoir workers, beef cattle, and abattoir environments in Abuja and Lagos. Stool, cecal, and environmental samples were collected from apparently healthy workers, slaughtered cattle, and abattoir environments from May to December 2020. Data were collected electronically using open data kit app installed on a mobile phone. Antimicrobial susceptibility patterns were determined using the Kirby–Bauer disk diffusion method against a panel of 16 antimicrobial agents. Phenotypic and genotypic characterizations of the isolates were conducted. Data were analyzed with descriptive statistics.

**Results:**

From 21.7% (*n* = 97) of 448 samples, ESBL-EC were isolated and further characterized. Prevalence of ESBL-EC was highest in cattle (45.4%; *n* = 44), abattoir workers (41.2%; *n* = 40), and abattoir environment (13.4%; *n* = 13). Whole-genome sequencing of ESBL-EC showed dissemination of *bla*CTX-M-15 (90.7%; *n* = 88); *bla*CTX-M-14 (5.2%; *n* = 5); and *bla*CTX-M-55 (2.1%; *n* = 2) genes. The *bla*CTX-M-15 coexisted with *bla*CTX-M-14 and *bla*TEM-1 genes in 2.1% (*n* = 2) and 39.2% (*n* = 38) of the isolates, respectively. The presence of *bla*CTX-M-14 and *bla*CTX-M-15 genes was significantly associated with isolates originating from abattoir workers when compared with beef cattle isolates (*p* = 0.05; *p* < 0.01). The most prevalent sequence types (ST) were ST10 (*n* = 11), ST215 (*n* = 7), ST4684 (*n* = 7), and ST2178 (*n* = 6). ESBL-EC strain (ST*205*/*B1*) harbored *mcr*-1.1 and *bla*CTX-M15 and was isolated from a worker at Lagos abattoir. In 91 ESBL-EC isolates, 219 mobile genetic elements (MGEs) harbored resistance genes out of which β-lactam genes were carried on 64 different MGEs. Isolates showed equal distribution of insertion sequences and miniature inverted repeats although only a few composite transposons were detected (humans *n* = 12; cattle *n* = 9; environment *n* = 4). Two isolates of human and cattle origin (ST46/A) harboring ESBL genes and carried by MGEs were clonally related.

**Conclusions:**

This is the first report of *bla*CTX-M-55 gene in humans and cattle in Nigeria. This study demonstrates the horizontal transfer of ESBL genes possibly by MGEs and buttresses the importance of genomic surveillance. Healthcare workers should be sensitized that people working closely with cattle or in abattoir environments are a high-risk group for fecal carriage of ESBL-EC when compared with the general population.

## Introduction

Antimicrobial-resistant food-borne bacteria have become an emerging problem in both human and animal populations (Rahman et al., 2017; Pormohammad et al., 2019; Ramos et al., 2020). The global spread of drug-resistant Enterobacterales has been associated with blood stream and urinary tract infections in humans (Runcharoen et al., 2017). During treatment of Gram-negative bacterial infections, an important resistance mechanism observed is the reduction in the efficacy of cephalosporins and monobactams (Geser et al., 2012). This is achieved by producing enzymes capable of inactivating these antimicrobial agents by hydrolyzing their beta-lactam ring. Extended-spectrum beta-lactamase (ESBL) efficiently hydrolyze 3rd- and 4th-generation cephalosporins with negative impact on patient care resulting in increased length of hospital stay, high morbidity, mortality, and high treatment costs (Geser et al., 2012; Runcharoen et al., 2017). ESBL genes were previously detected on chromosomes; however, these genes are now harbored on plasmids with resultant derivatives of plasmid-mediated β-lactamases such as *bla*TEM including *bla*CTX-M derived from the environment (Overdevest et al., 2011; Mensah et al., 2016).

The first description of ESBL production among Enterobacterales was from hospitalized patients although in recent years there has been reports of community-acquired ESBL infections (Reist et al., 2013; Song et al., 2020). There have also been increased reports of ESBL-producing Enterobacterales in humans, food-producing animals, and the environment in many countries (Reist et al., 2013; Rahman et al., 2017; Dantas Palmeira and Ferreira, 2020; Ramos et al., 2020; Song et al., 2020) although most bacteremias observed in humans may not be associated with animal reservoirs (Day et al., 2019). This has been attributed to the growing reservoirs as well as the indiscriminate use of antimicrobials (Overdevest et al., 2011; Veenemans et al., 2014). The ESBL-EC-Tricycle project uses a model that targets monitoring the indicator bacteria across the human, animal, and environmental sectors (WHO, 2021). Our recently published work showed that ESBL-EC, a zoonotic pathogen was detected among apparently healthy poultry workers, chickens, and the poultry farm/market environment (Aworh et al., 2020).

Food-producing animals including cattle have been reported as potential reservoirs for the spread of ESBL-EC along the food chain (Overdevest et al., 2011; Nguyen et al., 2019; Falgenhauer et al., 2019). Beef cattle, one of the food-producing animals, are linked to humans through shared environment and the food chain as a major source of animal protein (Marshall and Levy, 2011). In Nigeria like many developing economies, many families depend on beef cattle production as a means of livelihood as well as a major source of animal protein, hence the increased consumption of beef (Bettencourt et al., 2015). One of the gaps identified from an antimicrobial resistance (AMR) and use situation analysis across human, animal, and environmental health was the nonavailability of studies in Nigeria on ESBL-EC using a One Health approach (Nigeria Centre for Disease Control, 2017; Aworh et al., 2019). It is therefore imperative to assess the impact of healthy cattle as a possible reservoir for ESBL-EC along the food chain and to humans.

We hypothesized that slaughtered beef cattle harboring ESBL-EC can become potential sources of transmission of resistant genes along the food chain or to abattoir workers exposed based on their occupation as well as to the abattoir environments. We investigated the prevalence of fecal carriage of ESBL-EC among abattoir workers, beef cattle, and the abattoir environment to better understand if an association exists using antimicrobial susceptibility testing and whole-genome sequencing.

## Materials and Methods

### Study Design and Sample Collection

This cross-sectional study was carried out in one abattoir each in two major cities—Abuja (North/dry) and Lagos (South/humid), Nigeria, from May 2020 to December 2020. Using sterile stool containers, we collected freshly passed stool from apparently healthy consenting abattoir workers who were randomly selected. Cecal contents were randomly collected from the cecum of slaughtered beef cattle using sterile universal containers. For this study, we did not have access to information on animal husbandry and antimicrobial use in the slaughtered animals. Environmental samples comprising 30 g of lairage litter, 100 ml of abattoir waste water, and meat stall swabs were randomly collected from different locations in the selected abattoirs using sterile containers. All samples were transported in cool boxes to the National Reference Laboratory, Nigeria Centre for Disease Control Gaduwa, Abuja and processed for the presence of ESBL-EC.

### Isolation and Identification of ESBL-EC Isolates

We modified the WHO ESBL-Tricycle project protocol for the detection of ESBL-producing *E. coli* for the study (WHO, 2021). Briefly, about 1 g of human stool sample, 1 g of cecal content, and 30 g of litter samples were inoculated respectively in enrichment broth (buffered peptone water) in a 1:10 sample to broth ratio and incubated at 37°C for 24 h. Subsequently, a 10-µl loop-full of overnight culture from enrichment broth was plated onto MacConkey agar supplemented with cefotaxime (1 mg/L) and incubated at 37°C for 24 h. Next, suspected pink ESBL-EC colonies were further streaked on eosin methylene blue agar and incubated at 37°C for 24 h. Colonies suggestive of ESBL-EC were confirmed biochemically using commercially available Microbact GNB 24E (Oxoid, UK) according to the manufacturer’s instructions.

### Isolation of ESBL-EC From Waste Water Samples

We used the membrane filtration technique for the isolation of ESBL-EC from abattoir waste water samples. Each 100 ml of waste water sample was filtered using single sterile 0.45 μm pore filter disks placed in a filtration unit. Thereafter, the filter membranes were placed on MacConkey agar supplemented with cefotaxime (1 mg/L) and incubated at 37°C for 24 h. All suspected ESBL-EC isolates were further tested as previously described.

### Antimicrobial Susceptibility Testing

Antimicrobial susceptibility profiling of suspected ESBL-EC was conducted and interpreted according to the 2020 Clinical and Laboratory Standards Institutes (CLSI) M100 30th Edition recommendations (Clinical and Laboratory Standards Institute, 2020). The Kirby–Bauer disk diffusion method was used to determine the resistance profile of the ESBL-EC against a panel of 16 antimicrobial agents characterized by WHO to be useful in clinical case management and livestock production (WHO, 2019). Four of these were β-lactam antimicrobials comprising ampicillin, ceftazidime, cefotaxime, and cefoxitin while the remaining were non-β-lactam antimicrobials (azithromycin, chloramphenicol, ciprofloxacin, colistin, gentamicin, imipenem, meropenem, nalidixic acid, nitrofurantoin, sulphonamide, trimethoprim, and tetracycline). Briefly, one to two distinct colonies picked from the overnight culture were suspended into 5 ml of sterile saline solution with turbidity adjusted to 0.5 McFarland standard. Using a sterile cotton swab, the suspension was emulsified onto Mueller Hinton agar plate and incubated at 37°C for 18 h. Thereafter, the zone of inhibition was measured and the results were interpreted based on CLSI recommendations (Clinical and Laboratory Standards Institute, 2020). We used *E. coli* ATCC 25922 and *K. pneumoniae* strain ATCC 700603 for internal quality control. An ESBL-EC isolate observed to be resistant to three or more classes of antimicrobials was considered to be multidrug resistant (MDR).

### Detection of ESBL Phenotype by Disk Diffusion Test

ESBL production was confirmed phenotypically using the combination disk diffusion method as described by CLSI M100 30th Edition (Clinical and Laboratory Standards Institute, 2020). The confirmed ESBL-EC isolates had an increase in zone diameter of ≥5 mm for either cefotaxime or ceftazidime in combination with clavulanic acid when compared with either of the cephalosporin alone. We used *E. coli* ATCC 25922 and *K. pneumoniae* ATCC 700603 strains as negative and positive controls, respectively (Clinical and Laboratory Standards Institute, 2020).

### Whole Genome Sequencing of ESBL-EC

Whole genome sequencing was performed at the Noguchi Memorial Institute for Medical Research, University of Ghana under the SEQAFRICA Project. Briefly, the DNA for all ESBL-EC isolates from overnight culture were extracted and purified using Qiagen Kit following the manufacturer’s instructions. This was followed by quantification of DNA concentrations using the Qubit 4.0 Fluorometer Assay Kit (Thermo Fisher Scientific, MA, USA). Thereafter, libraries were prepared using the Nextera Flex Kit according to the manufacturer’s instructions. The libraries were quantified using the 2100 Bioanalyzer System (Agilent) and Kapa Sybr Fast qPCR Kit. DNA from each isolate were pooled together and sequenced on an Illumina Miseq platform using a 2 × 300 paired-end approach (Illumina Inc., San Diego, CA, USA). Raw sequencing reads (fastq files) were quality filtered to a Phred score ≥20, filtered for a minimum read length of 50 bp, and adaptor trimmed using Trimmomatic (http://www.usadellab.org/cms/index.php?page=trimmomatic). FastQC tool was used to assess quality of reads (https://www.bioinformatics.babraham.ac.uk/projects/fastqc/). The resultant high-quality reads were used for *de novo* assembly using the Unicycler assembler v0.4.9 (Wick et al., 2017).

### *In Silico* Detection of AMR Genes and Multilocus Sequence Typing (MLST)

The resistance genes were detected *in silico* using ResFinder (database version 2021-09-03) with identity threshold and minimum length set at 90% and 60%, respectively (Bortolaia et al., 2020). Genes with the highest sequence identity and coverage were retained while genes that overlapped were filtered out. Plasmid replicon types of each isolate was determined using Plasmid-Finder 2.1 (database version 2020-07-01) (Carattoli et al., 2014). The mobile genetic elements (MGEs) were predicted using MobileElementFinder (database version 2020-06-09) (Johansson et al., 2021). Multilocus sequence typing was conducted using MLSTFinder (database version 2021-10-18) with multilocus sequence typing (MLST) allele sequence and profile data from PubMLST.org which assigned sequence types (STs) based on allelic variations to seven housekeeping genes (*adk*, *fumC*, *gyrB*, *icd*, *mdh*, *purA*, and *recA*) that matched 100% identity (Larsen et al., 2012).

### Mobile Genetic Elements Associated With AMR

Each resistance gene was classified as being carried by a MGE or having no association. The resistance gene was associated if it was located on a MGE. The integrating MGEs associated with resistance genes were grouped on the basis of MGE type.

### Phylogenetic Analysis

The ESBL-EC isolates were assigned phylogenetic groups using *in silico* ClermonTyping 1.4.1 tool (Beghain et al., 2018). We mapped assembled genome contigs to *E. coli* reference genome (GenBank accession No. GCA_900636075.1) and constructed a maximum likelihood phylogenetic tree using CSI phylogeny tool from the Center for Genomic Epidemiology (CGE). Clonal relationship between ESBL-EC isolates was determined using pairwise single-nucleotide polymorphism (SNP) analysis in the *E. coli* core genome if two or more isolates had less than 30 different SNPs. However, isolates with fewer than 10 different SNPs were considered closely related. The SNP-based phylogenetic tree was visualized using the iTOL tool.

### Data Collection and Analyses

Electronic-structured questionnaires were administered using the open data kit (ODK) collect app which was installed on a mobile phone. Data were extracted offline onto a computer using ODK briefcase. Analysis was done by computing frequencies, proportions, and Chi-square test with a significance level of 0.05. The raw sequencing reads data for this study have been deposited in the National Center for Biotechnology Information (NCBI) under the Bio project accession number PRJNA797451.

### Ethics Approval and Consent to Participate

Approval for study was obtained from the Scientific and Ethical committee of the FCT Health Research Ethics Committee (Approval Number: FHREC/2020/01/40/04-05-20). Permission was sought from the management of each abattoir. Written informed consent was obtained from each eligible abattoir workers before questionnaire administration. We assured the abattoir workers of confidentiality of information obtained. We performed all the procedures according to the ethics committee’s guidelines and requirements.

## Results

### Prevalence of ESBL-EC in Humans, Cattle, and Abattoir Environments

Overall, 448 samples comprising human stool (*n* = 118), cattle cecal content (*n* = 272), and abattoir environment samples (*n* = 58) were collected from two large abattoirs in Abuja (*n* = 228) and Lagos (*n* = 220). The sample size for each abattoir varied depending on the actual size in terms of the number of cattle slaughtered daily and the willingness of the abattoir workers to participate in the study.

**Figure 1:**
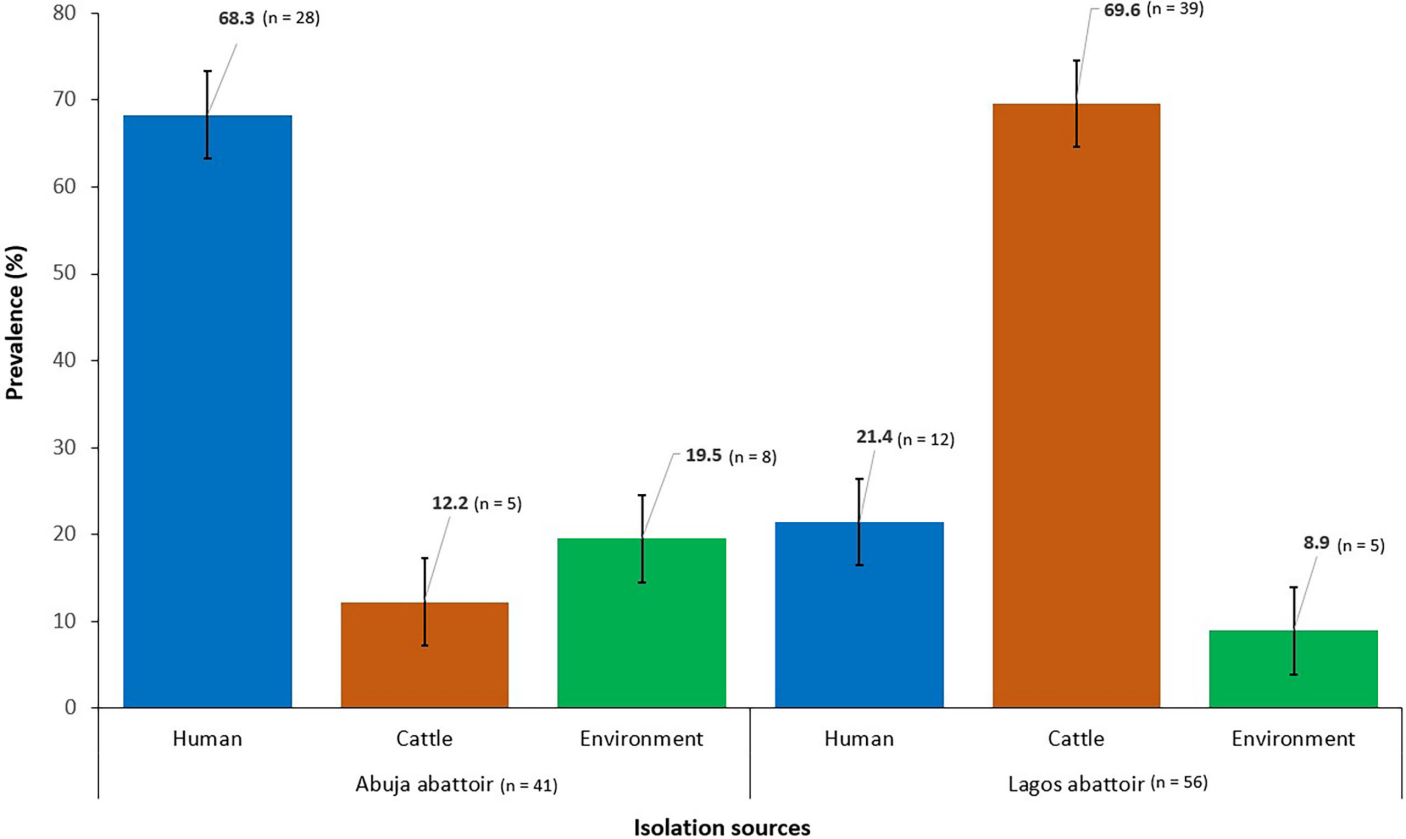
Prevalence of ESBL-EC isolated from humans, cattle, and abattoir environments in Abuja and Lagos Nigeria, 2021. Bars represent the proportion of ESBL-EC isolates from each isolation source with 95% confidence intervals. Error bars represent standard error of the mean prevalence. Data were obtained from two sources: Abuja and Lagos abattoirs.

The overall prevalence of ESBL-EC from all sources was 21.7% (*n* = 97) out of which 57.7% (*n* = 56) were obtained from Lagos abattoir and 42.3% (*n* = 41) from Abuja abattoir (**Figure 1**).

### Antimicrobial Susceptibility Profiling of ESBL-EC Isolates

Out of 97 ESBL-EC isolates, 99% (*n* = 96) were MDR. Out of these, 41.6% (*n* = 40) originated from abattoir workers, 44.8% (*n* = 43) were from cattle while 13.5% (*n* = 13) originated from the abattoir environment (**Table 1**). Overall, high resistance rates were observed in ESBL-EC to ceftazidime (89.7%), ciprofloxacin (95.9%), nalidixic acid (77.3%), tetracycline (82.5%), sulfonamides (78.4%), trimethoprim (76.3%), and azithromycin (52.6%), while low resistance rates were observed for gentamicin (19.6%) and chloramphenicol (18.6%). ESBL-EC isolates were however, susceptible to cefoxitin, imipenem, meropenem, and nitrofurantoin.

**Table 1:** Antimicrobial resistance profiles of ESBL-EC isolates from abattoir workers, beef cattle, and abattoir environments in Abuja and Lagos—Nigeria.

Drug class	Antimicrobial drugs	Resistance breakpoint (mm)	Humans (*n* = 40; %)	Cattle (*n* = 44; %)	Environs (*n* = 13; %)	Humans vs. cattle (*χ* ^2^; *p*)	Humans vs environs (*χ* ^2^; *p*)	Cattle vs. environs (*χ* ^2^; *p*)
**Tetracycline**	Tetracycline (30 μg)	<11	37 (92.5)	31 (70.5)	12 (92.3)	6.52 (**0.01**)	0.00 (0.98)	2.54 (0.11)
**Folate pathway antagonists**	Trimethoprim (5 μg)	<10	37 (92.5)	25 (56.8)	12 (92.3)	13.64 (**0.00**)	0.00 (0.98)	5.45 (**0.02**)
	Sulfonamides (300 μg)	<12	37 (92.5)	27 (61.3)	12 (92.3)	11.06 (**0.00**)	0.00 (0.98)	4.37 (**0.04**)
**Penicillins**	Ampicillin (10 μg)	<13	40 (100)	44 (100)	13 (100)	0.89 (0.35)	2.85 (0.09)	3.14 (0.08)
**Quinolones**	Nalidixic acid (30 μg)	<13	37 (92.5)	26 (59.1)	12 (92.3)	12.32 (**0.00**)	0.00 (0.98)	4.90 (**0.03**)
	Ciprofloxacin (5 μg)	<21	39 (97.5)	41 (93.2)	13 (100)	0.85 (0.36)	0.33 (0.57)	0.92 (0.34)
**Aminoglycosides**	Gentamicin (10 μg)	<12	12 (30)	2 (4.5)	5 (38.5)	9.66 (**0.00**)	0.32 (0.57)	10.53 (**0.00**)
**Phenicols**	Chloramphenicol (30 μg)	<12	12 (30)	4 (9.1)	2 (15.4)	5.87 (**0.02**)	1.06 (0.30)	0.41 (0.52)
**Macrolides**	Azithromycin (15 μg)	<12	10 (25)	39 (88.6)	2 (15.4)	34.49 (**0.00**)	0.51 (0.48)	26.20 (**0.00**)
**Nitrofurans**	Nitrofurantoin (300 μg)	<14	0 (0)	0 (0)	0 (0)	15.67 (**0.00)**	3.35 (0.07)	0.82 (0.37)
**Carbapenems**	Imipenem (10 μg)	<19	0 (0)	0 (0)	0 (0)	15.67 (**0.00)**	3.35 (0.07)	0.82 (0.37)
	Meropenem (10 μg)	<19	0 (0)	0 (0)	0 (0)	15.67 (**0.00)**	3.35 (0.07)	0.82 (0.37)
**Cephalosporins**	Cefotaxime (30 μg)	<22	40 (100)	44 (100)	13 (100)	0.89 (0.35)	2.85 (0.09)	3.14 (0.08)
	Ceftazidime (30 μg)	<17	36 (90)	39 (88.6)	12 (92.3)	0.04 (0.84)	0.06 (0.81)	0.14 (0.71)
	Cefoxitin (30 μg)	<14	0 (0)	0 (0)	0 (0)	15.67 (**0.00)**	3.35 (0.07)	0.82 (0.37)
**Resistance to 3 or more classes of antibiotics**	MDR	n/a	40 (100)	43 (97.7)	13 (100)	0.91 (0.34)	2.85 (0.09)	0.30 (0.59)

χ^2^, Chi-square; p, p-value; n, total number. Bold means statistically significant p-values.

### ESBL Genes in Abattoir Workers, Beef Cattle, and Abattoir Environments

Among ESBL producers, the *bla*CTX-M gene was the most predominant ESBL encoding gene detected in 96.9% (*n* = 94) of isolates from the various sources. The *bla*CTX-M-15, *bla*CTX-M-14, and *bla*CTX-M-55 genes were observed in 90.7% (*n* = 88), 5.2% (*n* = 5), and 2.1% (*n* = 2) ESBL-EC isolates, respectively. The *bla*CTX-M-14 coexisted with *bla*CTX-M-15 gene in two isolates originating from beef cattle and abattoir environment. None of the isolates were observed to carry any carbapenemase gene. Most 84.5% (*n* = 82) of the isolates carried the plasmid-mediated quinolone-resistant (PMQR) gene—*qnrS1*. Interestingly, one isolate of human origin was observed to carry plasmid-mediated colistin-resistant (PMCR) gene—*mcr 1.1*. The presence of *bla*CTX-M-14 and *bla*CTX-M-15 genes were significantly associated with isolates originating from abattoir workers when compared with beef cattle isolates (*p* = 0.05; *p* < 0.01) as shown in **Table 2**.

**Table 2:** ESBL gene variants of isolates originating from abattoir workers, beef cattle, and abattoir environments.

ESBL genes	Origin of isolates			H vs. C		H vs. E		C vs. E	
	Humans [*n* = 118; *n* (%)]	Cattle [*n* = 272; *n* (%)]	Environment [*n* = 58; *n* (%)]	*χ* ^2^	*p*	*χ* ^2^	*p*	*χ* ^2^	*p*
*bla*_CTX-M-14_	3 (2.54)	1 (0.37)	1 (1.72)	3.82	**0.05**	0.12	0.73	1.46	0.23
*bla*_CTX-M-15_	36 (30.5)	41 (15.07)	12 (20.68)	12.34	**0.00**	1.88	0.17	1.12	0.29
*bla*_CTX-M-55_	1 (0.85)	1 (0.37)	0 (0)	0.37	0.54	0.49	0.48	0.21	0.64

C, cattle; E, environment; H, human; χ^2^, Chi-square; p, p-value; n, total number. χ^2^, Chi-square; p, p-value; n, total number. Bold means statistically significant p-values.

### AMR Determinants Detected in ESBL-EC Isolates

**Figure 2:**
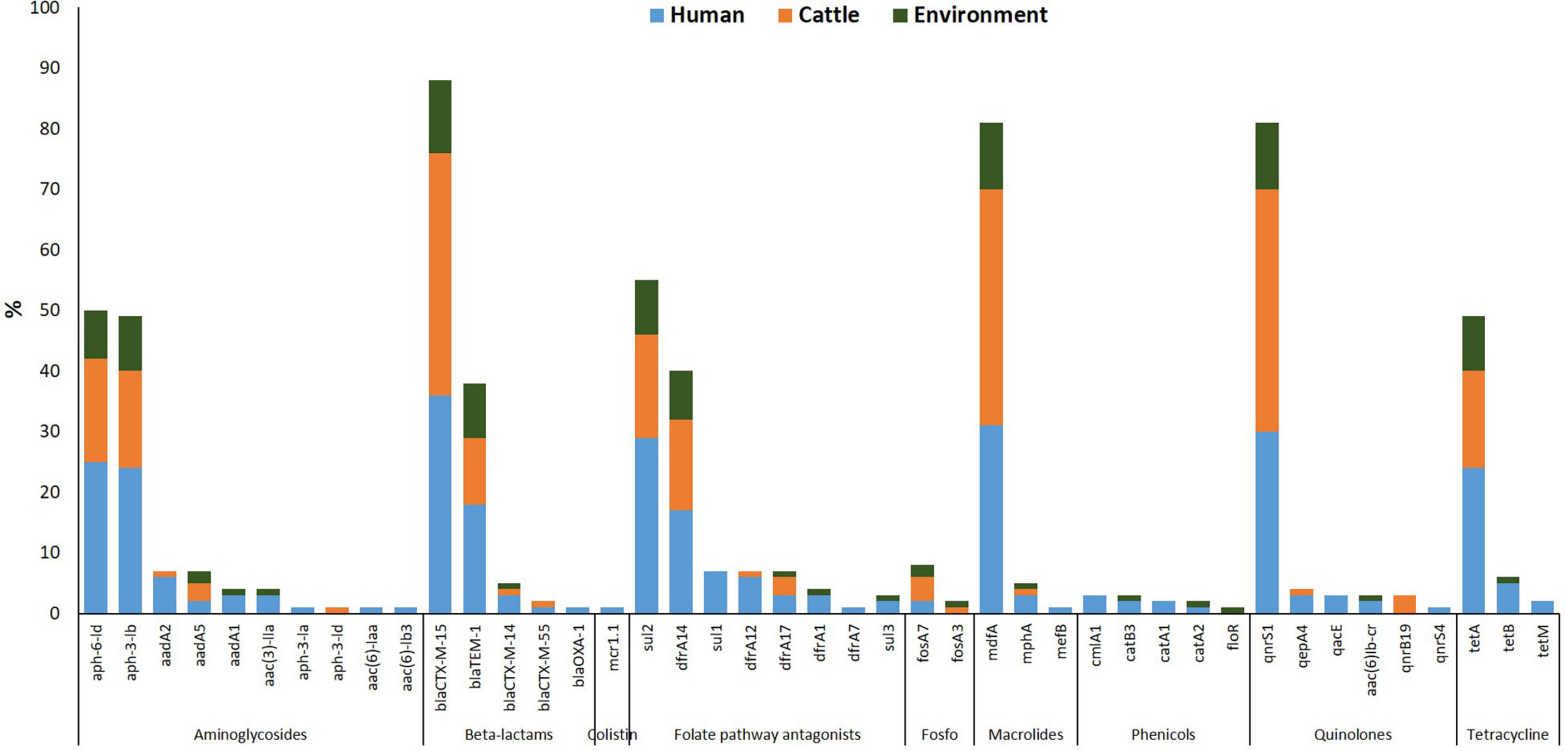
Resistance determinants detected in ESBL-EC isolates from humans, cattle, and abattoir environments in Abuja and Lagos—Nigeria. Each bar represents the various antimicrobial-resistant genes detected in ESBL-EC isolates obtained from abattoir workers, beef cattle, and abattoir environments.

Forty-seven different AMR determinants were identified in 97 ESBL-EC isolates with the most prevalent displayed in **Figure 2**. Aminoglycosides were the most detected with 11 different variants (*aadA1*, *aadA2*, *aadA5*, *aac(3)-Ila*, *aac(6)-Iaa*, *aac(6)-Ib3*, *aac(6)-Ib-cr*, *aph(3)-Ia*, *aph(3)-Ib*, *aph(3)-Id*, and *aph(6)-Id*). Three ESBL-EC isolates exhibited the *aac(6)-Ib-cr* gene responsible for the reduction in ciprofloxacin activity. The *aph(6)-Id* a plasmid-encoded gene was detected in more than half of the isolates. This was followed by the detection of folate pathway antagonists with eight different variants (*sul1*, *sul2*, *sul3*, *dfrA1*, *dfrA7*, *dfrA12*, *dfrA14*, and *dfrA17*). The β-lactam genes were of five different variants (*bla*TEM-1, *bla*OXA-1, *bla*CTX-M-14, *bla*CTX-M-15, and *bla*CTX-M-55) out of which *bla*CTX-M variant was classical of ESBL-EC. Quinolone-resistant genes often associated with chromosomal mutations and categorized as critically important antimicrobials by the World Health Organization were detected in ESBL-EC isolates with six different variants (*aac(6)-Ib-cr, qnrB19*, *qnrS1*, *qnrS4*, *qepA4*, and *qacE*). Other AMR determinants detected in ESBL-EC isolates were polymyxin resistance (plasmid-mediated colistin resistance *mcr*-1.1), phenicol resistance (*catA1*, *catA2*, *catB3*, *cmlA1*, *floR*), fosfomycin resistance (*fosA3*, *fosA7*), macrolide resistance (*erm(B)*, *mef(B)*, *mph(A)*, *mdfA)*, and tetracycline resistance (*tet*A, *tet*B, *tet*M).

### Multilocus Sequence Determination of ESBL-EC Isolates

**Figure 3:**
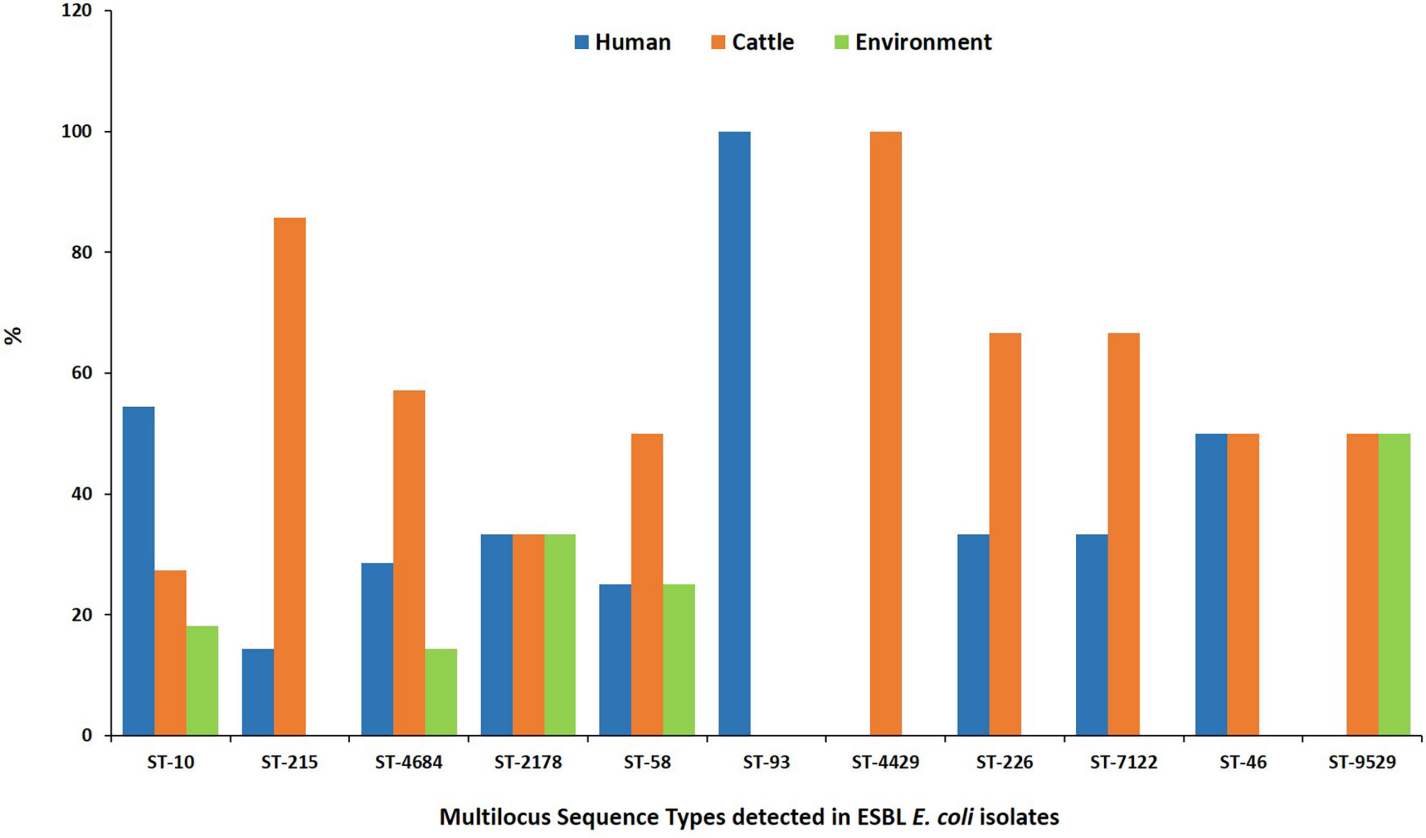
Multilocus sequence types for ESBL-EC isolated from humans, beef cattle, and abattoir environments in Abuja and Lagos—Nigeria. Each bar represents the various ESBL-EC sequence types for isolates obtained from abattoir workers, beef cattle, and abattoir environments.

MLST analysis showed that 97 ESBL-EC isolates were assigned to 52 known sequence types (STs). Four STs being ST 10, ST 2178, ST 4684, and ST 58 were shared by 28.9% (*n* = 28) of isolates from all three sources (**Figure 3**). Four STs were shared between abattoir workers and beef cattle (ST 46, ST 226, ST 7122, and ST 215) while ST 9529 was shared between ESBL-EC isolates from beef cattle and the abattoir environment. The most common ESBL variant detected was *bla*CTX-M-15 (90.7%). All *bla*CTX-M-15 isolates belonged to 49 different STs with eight being prevalent: ST 10 (*n* = 11), ST 215 (*n* = 7), ST 4684 (*n* = 7), ST 2178 (*n* = 6), ST 58 (*n* = 4), ST 226 (*n* = 3), ST 4429 (*n* = 3), and ST-7122 (*n* = 3).

### Association of MGEs With AMR

**Figure 4:**
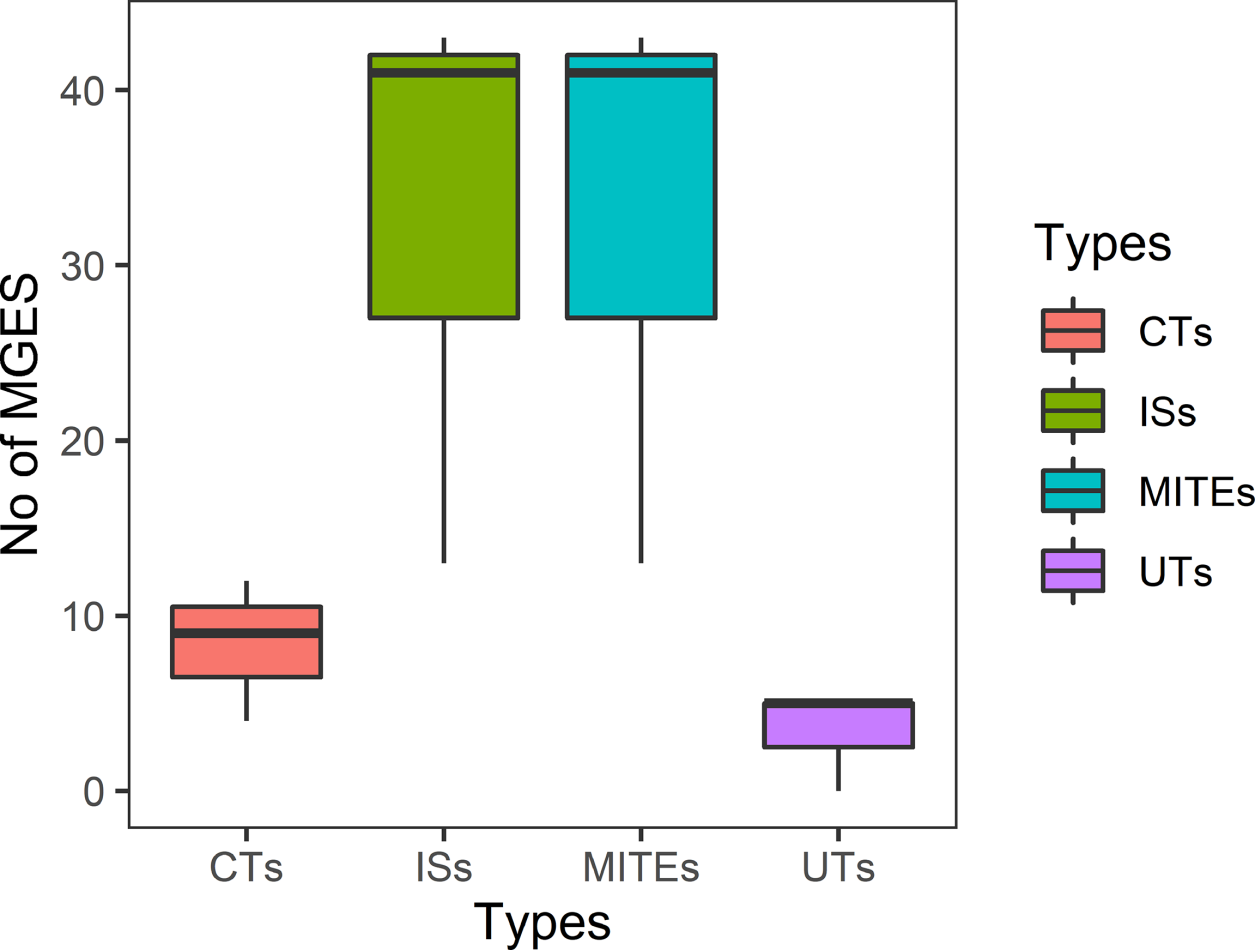
Total number of predicted MGEs in ESBL-EC isolates from all sources.

Isolates from all sources showed equal distribution of insertion sequences (ISs) and miniature inverted repeats (MITEs). Of 97 ESBL-ECs, 25.8% (*n* = 25) were composite transposons (CTs) while 10.3% (*n* = 10) were unit transposons (UTs). Of 25 CTs, 48% (*n* = 12), 36% (*n* = 9), and 16% (*n* = 4) were detected in isolates from humans, cattle, and abattoir environments respectively with 52% from Lagos and 48% from Abuja abattoirs. The 10 UTs were detected in equal proportions between ESBL-EC isolates from humans and cattle. In 91 ESBL-EC isolates, 219 MGEs were observed to harbor AMR genes out of which β-lactam genes were carried on 64 different MGEs. Of these, 31 (48.4%) carried *bla*CTX-M-15 + *qnrS1*; 29 (45.3%) carried *bla*CTX-M-15; 2 (3.1%) carried *bla*CTX-M-55 + *qnrS4* while 2 (3.1%) carried *bla*CTX-M-55 + *qnrS4.*The β-lactam genes were more frequently associated with MGEs in isolates from beef cattle (67.2%) than those from abattoir workers (23.4%) and the abattoir environment (9.4%). Eight different types of ISs and one MITE type were observed to harbor ESBL genes with ISEc9 (48.4%, *n* = 31), ISKpn19 (37.5%, *n* = 24), and MITTEc1 (7.8%, *n* = 5) being the most prevalent (**Figure 4**).

### Phylogenetic Analysis of ESBL-EC Isolates

**Figure 5:**
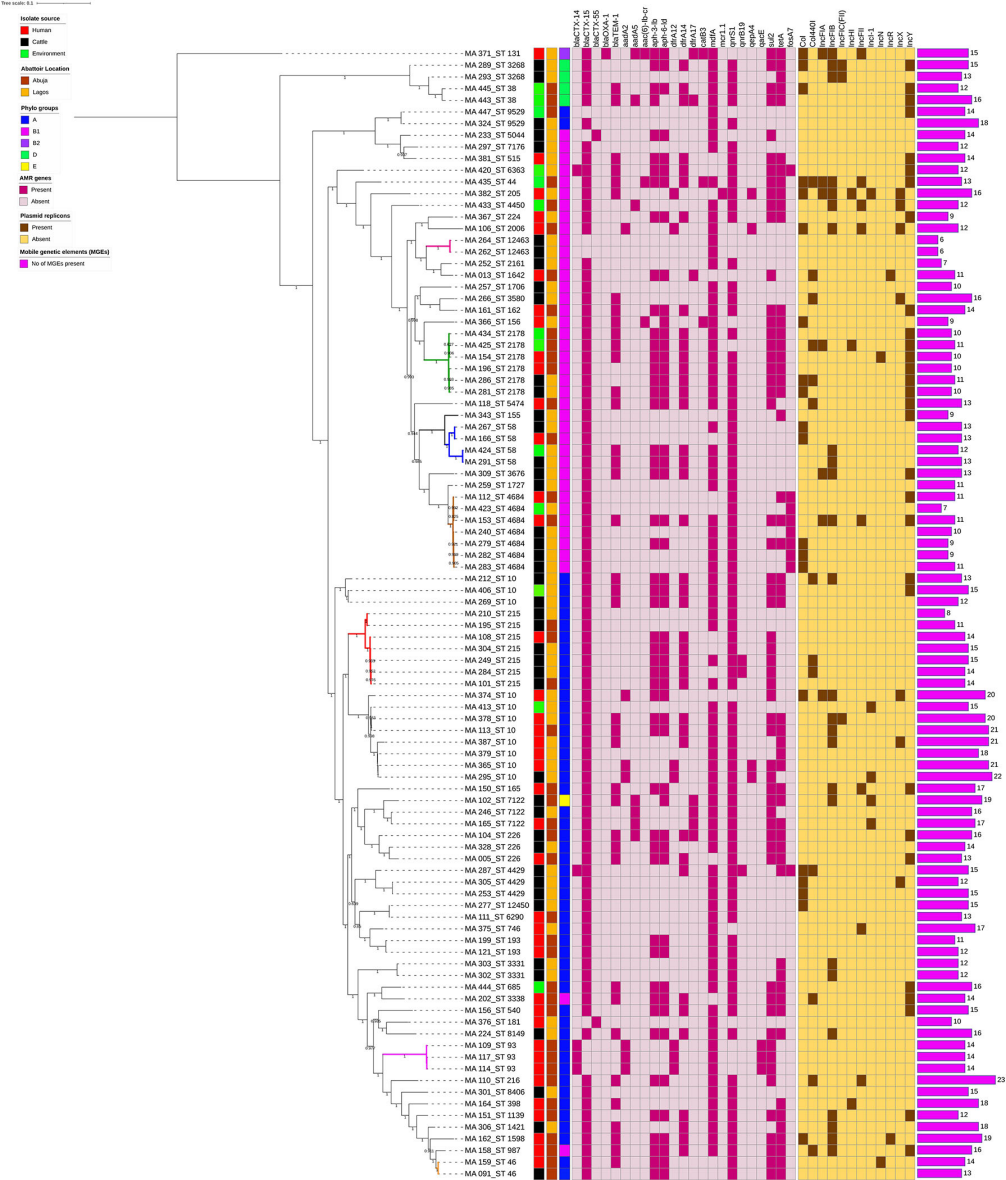
SNP-based phylogeny of ESBL *E. coli* isolates from humans, cattle, and abattoir environments in Abuja and Lagos. SNP-based maximum likelihood phylogeny of *E. coli* isolates visualized in iterative Tree of life tool (iTol). The tree was rooted in a reference isolate *E. coli* strain NCTC11129. Clustering of isolates was found to be following the core genome and SNP-based phylogenies. The clustering of isolates belonging to the same phylogenetic group and sequence type was consistent. Shown for each isolate is the source: humans, cattle or environment, location, phylogroup, sequence types (STs), AMR genes and plasmid replicons, and number of MGEs.

In the phylogenetic analysis, all isolates were assigned a phylo-group in descending order phylo-groups A (52.6%, *n* = 51), B1 (41.2%, *n* = 40), D (4.1%, *n* = 4), B2 (1%, *n* = 1), and E (1%, *n* = 1). Overall, 97 isolates were used to construct a SNP-based maximum likelihood phylogenetic tree (**Figure 5**). Only very few plasmid replicons were detected in the ESBL-EC isolates. It was evident that clonal relationship existed among isolates originating from different sources. ESBL-EC isolates were clustered into two main phylogroups and seven different STs. The pairwise distance matrix of SNPs based on core genome of 97 ESBL-EC strains showed 30 clonal relationships with a pairwise SNP difference of 30 out of which 11 (36.7%) were closely related with a pairwise SNP difference below 10. The clonally related isolates were clustered together based on the phylogenetic tree. Two isolates from the Abuja abattoir of human and cattle origin belonging to phylogroup A and sharing ST46 were observed to be closely related with a SNP difference of 2 (**Table 3**). In these two isolates, insertion sequences (ISKpn19 of ISKra4 family) were observed to carry the ciprofloxacin resistance gene (*qnrS1*). A combination of *qnrS1* and *bla*CTX-M-15 were carried by the insertion sequence in the cattle isolate.

**Table 3:** Clonal relationship between ESBL-EC isolates from different sources.

Clonal relationship	Sample ID	Species	SNP difference
**Isolates with pairwise distance of less than 10 SNPs**			
A	MA_091 and MA_159	Cattle and humans^a^	2
B	MA_267 and MA_166	Cattle and humans	2
C	MA_304 and MA_108	Cattle and humans	9
**Isolates with pairwise distance of less than 30 SNPs**			
D	MA_281 and MA_196	Cattle and humans	12
E	MA_249 and MA_108	Cattle and humans	14
F	MA_277 and MA_111	Cattle and humans	20
G	MA_154 and MA_434	Human and environ^a^	20
H	MA_281 and MA_154	Cattle and humans	25
I	MA_153 and MA_423	Human and environ	25
J	MA_154 and MA_425	Human and environ^a^	25
K	MA_284 and MA_108	Cattle and humans	26
L	MA_281 and MA_434	Cattle and environ	27
M	MA_295 and MA_365	Cattle and humans^a^	29
N	MA_196 and MA_434	Human and environ^a^	29
O	MA_101 and MA_108	Cattle and humans^a^	30

a Isolates from the same location.

## Discussion

Many studies globally have reported that *E. coli* isolated from food animals including beef cattle are often resistant to β-lactam antimicrobials (Tadesse et al., 2018; Ramos et al., 2020; Song et al., 2020; Dantas Palmeira and Ferreira, 2020). Cattle have been reported as an important source of ESBL-EC transmission to humans and a threat to the world (Sudarwanto et al., 2016; Saleem et al., 2017; Dantas Palmeira and Ferreira, 2020). The risk for people working in close proximity with animals has been poorly elucidated.

This present study investigated the prevalence of ESBL-EC among abattoir workers, beef cattle at slaughter, as well as in the abattoir environments. Our findings showed that ESBL-EC are present in the abattoir environment where beef cattle are processed for food serving as a reservoir of resistant pathogens and a health hazard to people working in such environment. The prevalence of ESBL-EC in the present study was 41.2%, 45.4%, and 13.4% in humans, beef cattle, and abattoir environments, respectively. Our study prevalence of ESBL-EC in cattle was higher than what was observed in another location in Nigeria but much lower than the prevalence reported among abattoir workers (Egbule and Yusuf, 2019; Egbule and Iweriebor, 2021). Similar studies in Europe reported a much lower prevalence of ESBL-EC in cattle and farm workers (Reist et al., 2013; Dahms et al., 2015). A possible explanation for the high prevalence observed in the present study may be as a result of over-the-counter availability of antimicrobials in Nigeria for both humans and animals as opposed to Europe where these are available based on prescription (Oluwasile et al., 2014; Oloso et al., 2018; Agyare et al., 2019).

Penicillin, cephalosporin, quinolones, tetracycline, folate pathway antagonists, and aminoglycosides accounted for most of the resistance determinants detected in the present study, and this is consistent with the reports of others (Reist et al., 2013; Oloso et al., 2018; Egbule and Yusuf, 2019; Kimera et al., 2020). Critically important antimicrobial-resistant genes PMQR—*qnrS1* and PMCR—*mcr 1.1* were detected in the ESBL-EC isolates. Our study detected higher resistance to ciprofloxacin when compared with nalidixic acid which is rather surprising. A possible explanation for this observation may be due to the high prevalence of *qnrS1.* The high prevalence of AMR genes observed may be attributed to the use of these antimicrobials in livestock production in Nigeria either for prophylaxis or therapeutic purposes (Oloso et al., 2018; Kimera et al., 2020).

The only ESBL gene variant detected in the present study was *bla*CTX-M in isolates from all sources, and this is consistent with findings from other studies (Dantas Palmeira and Ferreira, 2020; Kimera et al., 2020). The *bla*CTX-M-15 genes were the most prevalent and found to coexist with *bla*CTX-M-14 in two isolates from beef cattle and abattoir environment. Interestingly, *bla*CTX-M-55 gene was also detected in isolates from humans and cattle in this study. It is important to note that *bla*CTX-M-55 variant has been the second most predominant ESBL gene in Asia and has been reported in Europe but rare in Africa (Lupo et al., 2018; Dantas Palmeira and Ferreira, 2020).

Our study results showed evidence of genetic diversity of ESBL-EC isolates as we detected 52 known sequence types (STs), and this is consistent with reports of others (Day et al., 2019; Falgenhauer et al., 2019). The commonly detected STs and phylogroups in isolates originating from all sources were ST*10/A*, ST*2178/B1*, ST*4684/B1*, and ST*58/B1*, suggesting that a possible transmission may have occurred between these sources. This observation further buttresses that co-colonization of resistant foodborne bacteria from a shared source is possible. Evidence shows that *E. coli* ST*58* is an important zoonotic strain detected in food-producing animals particularly cattle implicated in the spread of ESBL-EC *t*o humans (Pietsch et al., 2018; Irenge et al., 2019). It is worthy of note that ST*10* has been detected in *E. coli* isolates from healthy humans, animals, and environmental samples (Müller et al., 2016; Falgenhauer et al., 2019), and this is consistent with our study results. Our observation may be as a result of a possible circulation of host-adapted lineages of ESBL-EC.

Although, some evidence exists that food-producing animals are important reservoirs in the transmission of resistant bacteria to the human population, our study clearly shows that there is a possibility. Several isolates originating from humans, cattle, and the abattoir environment demonstrated clonal relationship with 0–30 SNP differences between the isolates especially those with ST*12463*, ST*2178*, ST*58*, ST*4684*, ST*215*, ST*10*, ST*93*, and ST*46*. Our study demonstrated that one human and one cattle isolate (ST*46*) were closely related with a pairwise SNP difference of 2 suggesting a possibility of horizontal transfer of resistance between the two hosts and consistent with findings from a study in Ghana (Falgenhauer et al., 2019). Although the isolates in the Ghana study originated from chickens and sick children, our sources were apparently healthy cattle and abattoir workers.

In the MGE analysis, the MITEs and ISs were more predominant than the transposons in the ESBL-EC isolates in the present study and this observation is consistent in Gram-negative bacteria (Johansson et al., 2021). Majority of the resistance genes were not carried by transposons but by MITEs and ISs as reported by other studies (Partridge et al., 2018; Johansson et al., 2021). The two closely related isolates from humans and cattle originated from the same abattoir location, belonged to the same phylogroup, and had similar resistance genes carried by identical MITEs, ISs, and transposons. It is noteworthy that no ESBL genes were harbored on plasmid replicons.

To the best of our knowledge, this study is the first in Nigeria to report PMCR gene, *mcr*-1.1 in abattoir workers. The *mcr*-1.1-positive ESBL-EC strain (ST*205*/*B1*) coexisted with *bla*CTX-M-15 and was isolated from a worker at Lagos abattoir. Although the *bla*CTX-M-15 gene was carried on an insertion sequence, our results showed that the *mcr*-1.1 gene was not harbored by any MGE. Colistin which is considered a drug of last resort, is often used in livestock production in Nigeria for therapeutic purposes (Oluwasile et al., 2014; Aworh et al., 2019).

This study is not without limitations as we had very few people who agreed to participate in one of the abattoirs and may have been responsible for our observations.

ESBL-EC isolates were prevalent among apparently healthy abattoir workers, beef cattle, and the abattoir environment in Abuja and Lagos. Among ESBL-EC, the highest resistance was observed to ampicillin, cefotaxime, and ciprofloxacin, which are antimicrobial classes often used in livestock production for therapeutic purposes. Our study detected ESBL genes, *bla*_CTX-M_ in isolates from all the sources. The *bla*_CTX-M_ coexisted with *qnrS1* and *mcr*-*1.1* genes. To our knowledge, this is the first study in Nigeria to report *bla*_CTX-M-55_ gene in humans and cattle. The possibility of horizontal transfer of resistance genes from humans to cattle or vice versa may have been facilitated by MGEs. It is imperative to sensitize healthcare workers that abattoir workers or people working in such vicinities are a high-risk group for fecal carriage of ESBL-EC, hence pose a higher risk to the general population for horizontal transfer of resistance. The Government should develop and enforce regulations that prohibit the use of critically important antimicrobials for human health in food-producing animals.

## Data Availability Statement

The datasets presented in this study can be found in online repositories. The names of the repository/repositories and accession number(s) can be found in the article/**Supplementary Material**.

## Ethics Statement

The studies involving human participants were reviewed and approved by the FCT Health Research Ethics Committee (Approval Number: FHREC/2020/01/40/04-05-20). The patients/participants provided their written informed consent to participate in this study. The animal study was reviewed and approved by the Scientific and Ethical Committee of the Ahmadu Bello University Committee on Animal Use and Care (Approval Number: ABUCAUC/2020/35).

## Author Contributions

MA was the principal investigator, designed data collection tools, collected data, isolated the organism, performed antibiotic sensitivity testing on the isolates, analyzed and interpreted the data, and wrote the first draft of the manuscript. RH made substantial contributions to conception and design. EE, PN, and RH supervised the laboratory aspect of the research. BE and CO generated the whole genome sequence profile for all the isolates. MA performed bioinformatics analysis. EE, PN, BE, CO, and RH revised the article critically for important intellectual content. All authors read and approved the final manuscript.

## Funding

This project was funded by the Fleming Fund Fellowship scheme through the Denmark Technical University according to grant No. 13534.

## Conflict of Interest

The authors declare that the research was conducted in the absence of any commercial or financial relationships that could be construed as a potential conflict of interest.

## Publisher’s Note

All claims expressed in this article are solely those of the authors and do not necessarily represent those of their affiliated organizations, or those of the publisher, the editors and the reviewers. Any product that may be evaluated in this article, or claim that may be made by its manufacturer, is not guaranteed or endorsed by the publisher.

## Abbreviations

AMR, antimicrobial resistance; CLSI, Clinical and Laboratory Standards Institute; CTs, composite transposons; *E. coli*, *Escherichia coli*; ESBL, extended-spectrum beta-lactamase; ESBL-EC, extended-spectrum beta-lactamase-producing *E. coli*; ISs, insertion sequences; MDR, multidrug resistance; MIC, minimum inhibitory concentration; MITEs, miniature inverted repeats; MLST, multilocus sequence typing; MGEs, mobile genetic elements; NCBI, National Center for Biotechnology Information; ODK, open data kit; PMCR, plasmid-mediated colistin resistance; PMQR, plasmid-mediated quinolone resistance; SNP, single nucleotide polymorphisms; ST, sequence type; UT, unit transposons; WGS, whole-genome sequencing; WHO, World Health Organization.
