# Supplementary figures and images for: Extended-Spectrum ß-Lactamase-Producing Escherichia coli Among Humans, Beef Cattle, and Abattoir Environments in Nigeria
Source: Front Cell Infect Microbiol. 2022 Apr 7;12:869314. doi: 10.3389/fcimb.2022.869314 (PMC9021871; doi:10.3389/fcimb.2022.869314)

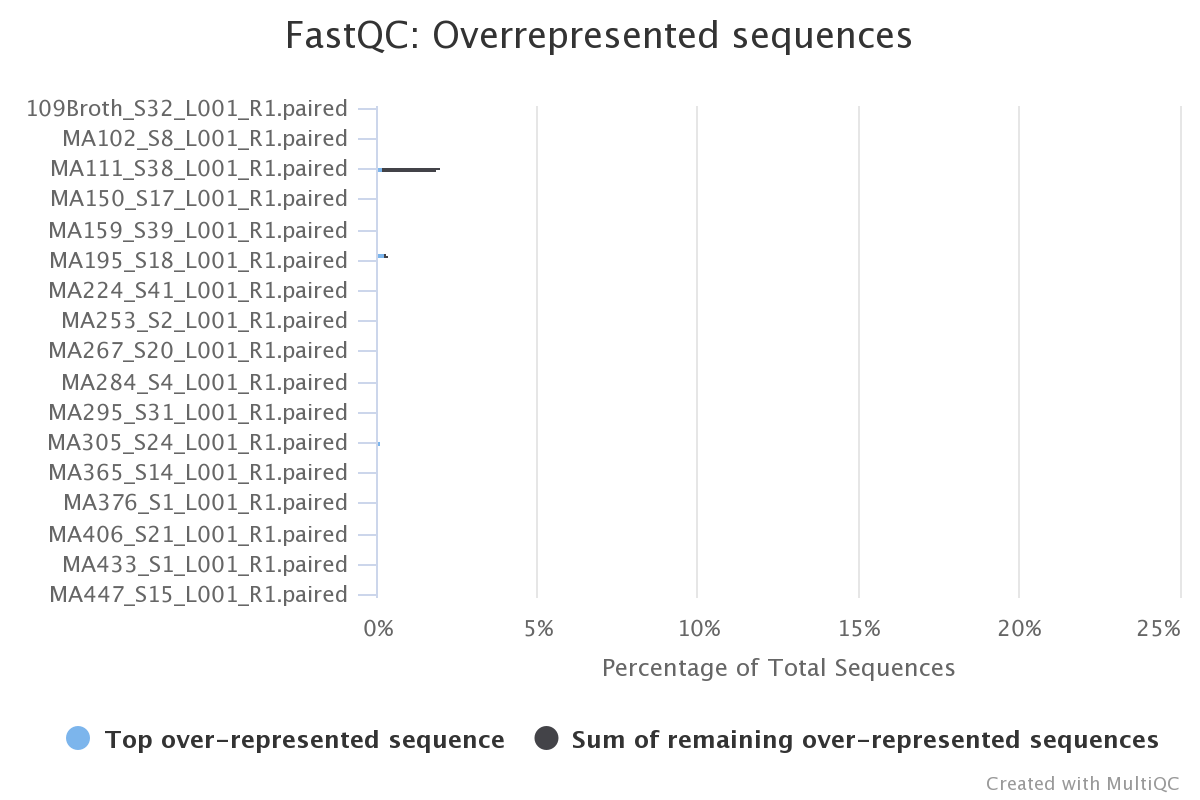

Supplement: Supplementary file 1 [file DataSheet_1.zip › Supplementary data/fastqc_overrepresented_sequences_plot.png]

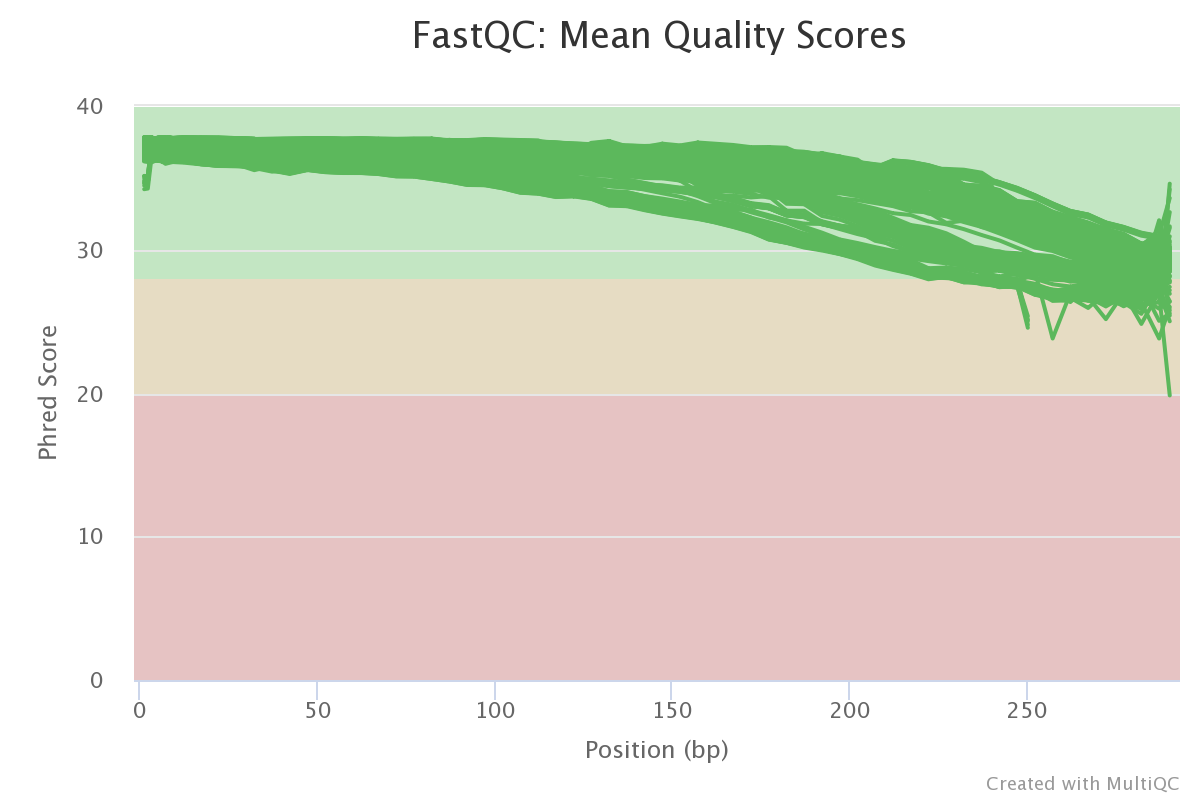

Supplement: Supplementary file 1 [file DataSheet_1.zip › Supplementary data/fastqc_per_base_sequence_quality_plot.png]

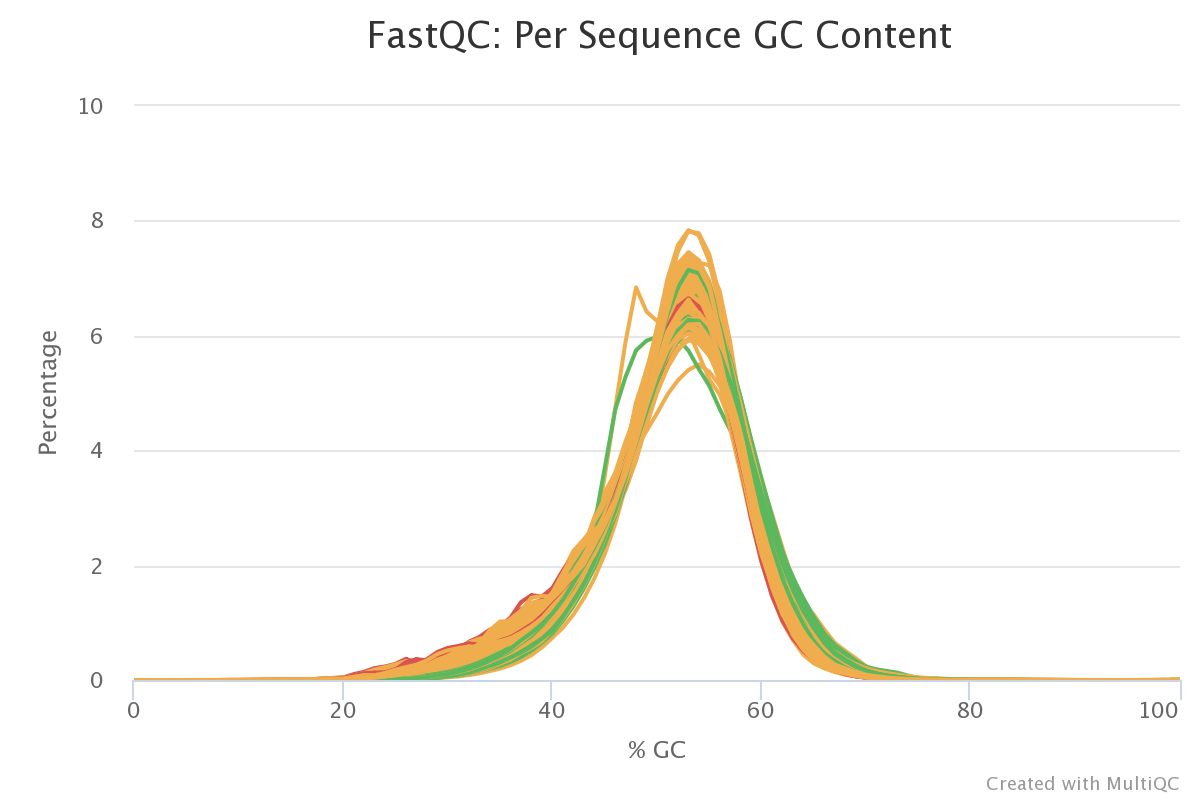

Supplement: Supplementary file 1 [file DataSheet_1.zip › Supplementary data/fastqc_per_sequence_gc_content_plot.png]

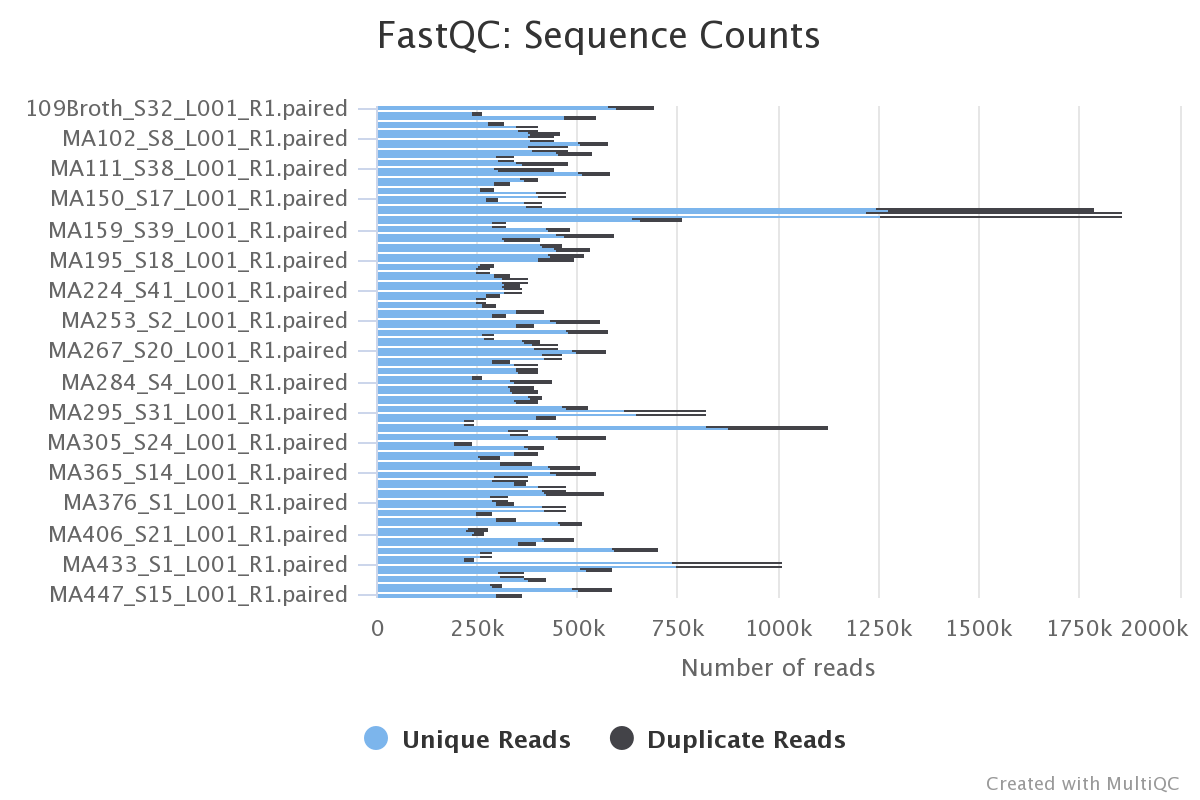

Supplement: Supplementary file 1 [file DataSheet_1.zip › Supplementary data/fastqc_sequence_counts_plot.png]

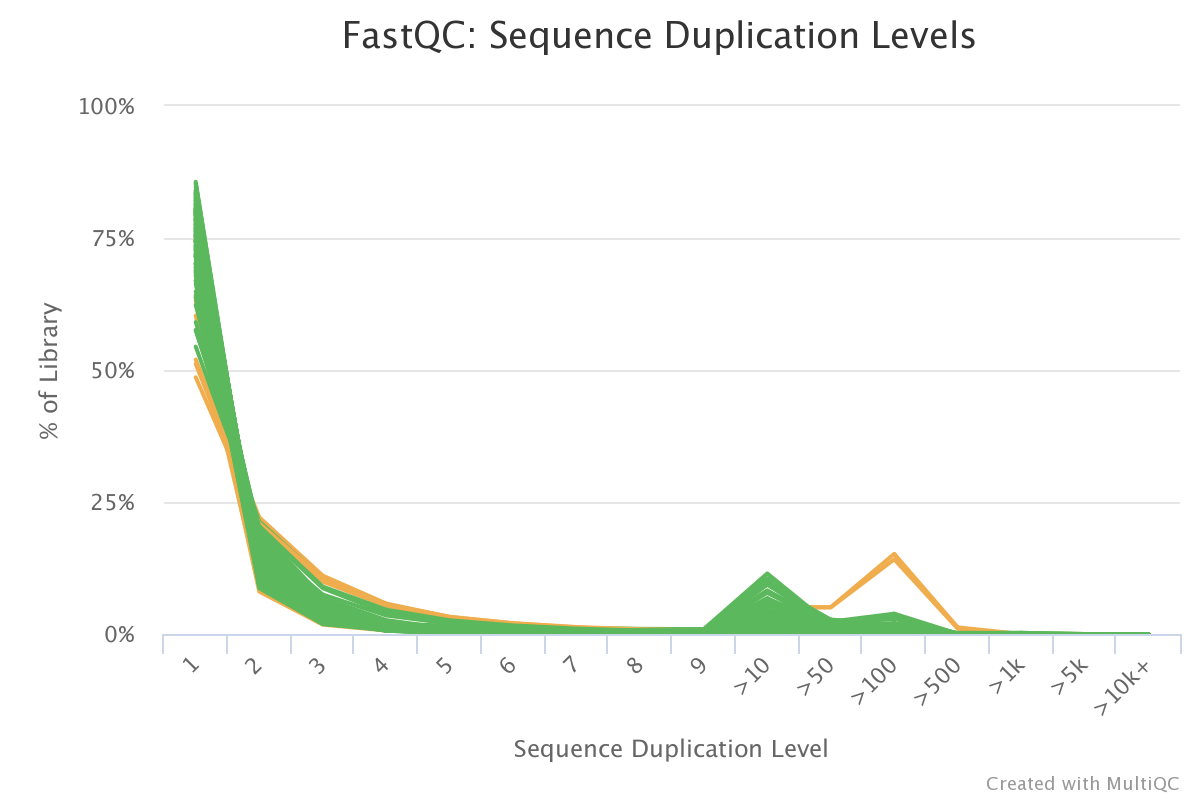

Supplement: Supplementary file 1 [file DataSheet_1.zip › Supplementary data/fastqc_sequence_duplication_levels_plot.png]

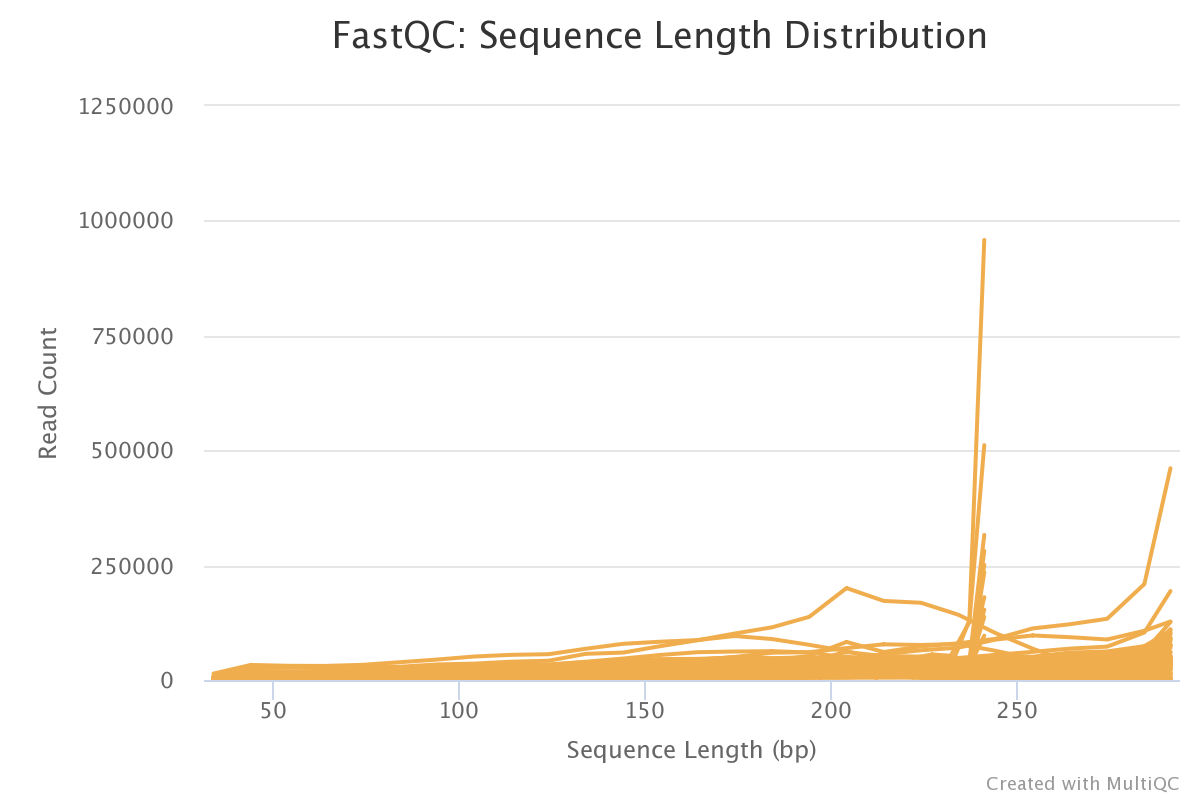

Supplement: Supplementary file 1 [file DataSheet_1.zip › Supplementary data/fastqc_sequence_length_distribution_plot.png]

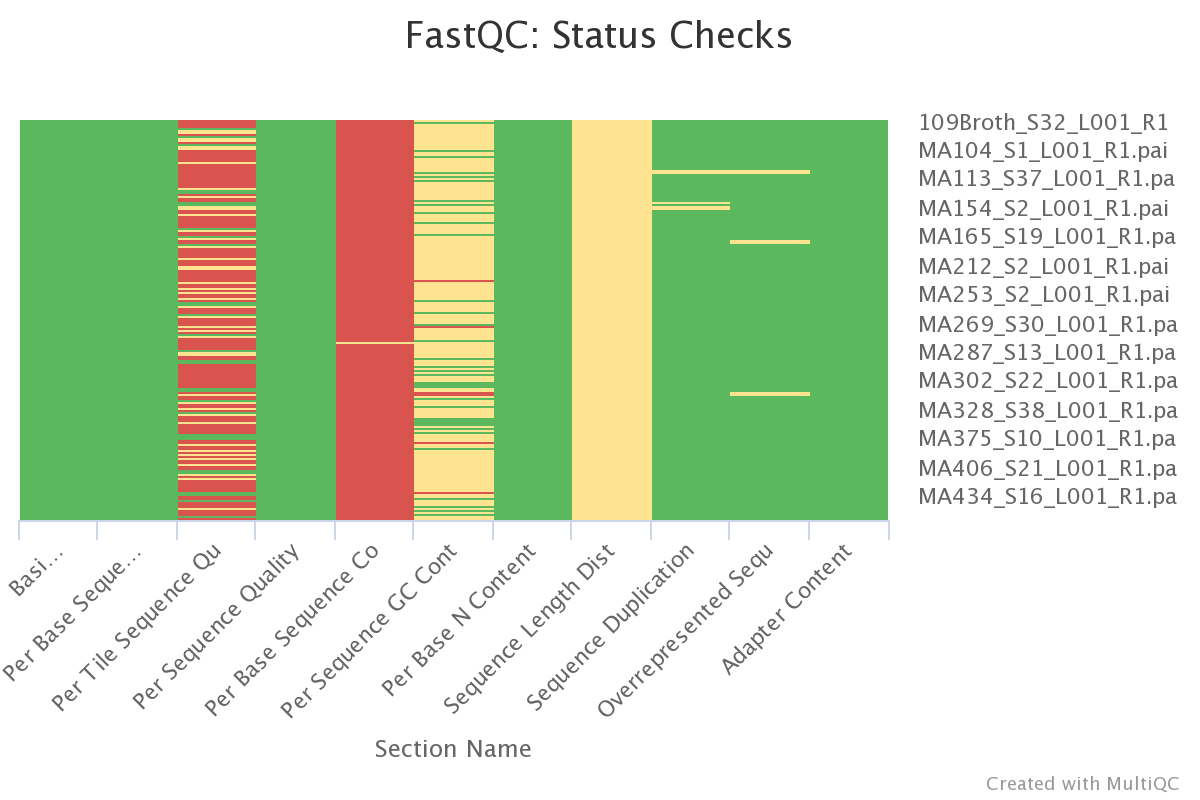

Supplement: Supplementary file 1 [file DataSheet_1.zip › Supplementary data/fastqc-status-check-heatmap.png]
